# Cell Adhesion Signaling Regulates RANK Expression in Osteoclast Precursors

**DOI:** 10.1371/journal.pone.0048795

**Published:** 2012-11-06

**Authors:** Ayako Mochizuki, Masamichi Takami, Yoichi Miyamoto, Tsuyoshi Nakamaki, Shigeru Tomoyasu, Yuho Kadono, Sakae Tanaka, Tomio Inoue, Ryutaro Kamijo

**Affiliations:** 1 Department of Biochemistry, School of Dentistry, Showa University, Shinagawa, Tokyo, Japan; 2 Department of Oral Physiology, School of Dentistry, Showa University, Shinagawa, Tokyo, Japan; 3 Division of Hematology, Department of Medicine, Showa University School of Medicine, Shinagawa, Tokyo, Japan; 4 Department of Orthopaedic Surgery, Faculty of Medicine, The University of Tokyo, Bunkyo, Tokyo, Japan; University of Lyon, France

## Abstract

Cells with monocyte/macrophage lineage expressing receptor activator of NF-κB (RANK) differentiate into osteoclasts following stimulation with the RANK ligand (RANKL). Cell adhesion signaling is also required for osteoclast differentiation from precursors. However, details of the mechanism by which cell adhesion signals induce osteoclast differentiation have not been fully elucidated. To investigate the participation of cell adhesion signaling in osteoclast differentiation, mouse bone marrow-derived macrophages (BMMs) were used as osteoclast precursors, and cultured on either plastic cell culture dishes (adherent condition) or the top surface of semisolid methylcellulose gel loaded in culture tubes (non-adherent condition). BMMs cultured under the adherent condition differentiated into osteoclasts in response to RANKL stimulation. However, under the non-adherent condition, the efficiency of osteoclast differentiation was markedly reduced even in the presence of RANKL. These BMMs retained macrophage characteristics including phagocytic function and gene expression profile. Lipopolysaccharide (LPS) and tumor necrosis factor –αTNF-α activated the NF-κB-mediated signaling pathways under both the adherent and non-adherent conditions, while RANKL activated the pathways only under the adherent condition. BMMs highly expressed RANK mRNA and protein under the adherent condition as compared to the non-adherent condition. Also, BMMs transferred from the adherent to non-adherent condition showed downregulated RANK expression within 24 hours. In contrast, transferring those from the non-adherent to adherent condition significantly increased the level of RANK expression. Moreover, interruption of cell adhesion signaling by echistatin, an RGD-containing disintegrin, decreased RANK expression in BMMs, while forced expression of either RANK or TNFR-associated factor 6 (TRAF6) in BMMs induced their differentiation into osteoclasts even under the non-adherent condition. These results suggest that cell adhesion signaling regulates RANK expression in osteoclast precursors.

## Introduction

Osteoclasts, multinucleated giant cells that resorb bone, differentiate from hematopoietic cells with a monocyte/macrophage lineage [1], [2], [3]. Osteoclast differentiation is supported by osteoblasts as well as stromal cells through cell-to-cell interactions [4], [5]. Osteoblasts produce the osteoclast differentiation factor receptor activator of NF-κB ligand (RANKL) (identical to TRANCE, ODF, OPGL, TNFSF11, and CD254) in response to several bone resorbing factors, such as 1α,25-dihydroxyvitamin D_3_ [1α,25(OH)_2_D_3_] and parathyroid hormone (PTH) [5], [6], [7]. Binding of RANKL to its receptor RANK on osteoclast precursors results in recruitment of TNF receptor-associated factor (TRAF) family proteins such as TRAF6, which activate NF-κB and MAP kinases (MAPKs). Such signaling subsequently activates the transcription factors c-fos, PU.1, and NFATc1, all of which are required for osteoclast differentiation [8], [9]. Among these factors, NFATc1 is selectively induced by stimulation with RANKL and functions as a master switch for regulating terminal differentiation of osteoclasts [10]. Disruption of RANKL or RANK results in osteopetrosis due to impaired osteoclast differentiation [11], [12], indicating that the RANK-RANKL system is essential for regulation of osteoclast differentiation *in vivo*.

It is generally accepted that not only RANKL, but also an adherent environment is required for osteoclast differentiation and functions [4], [5]. Some studies have found that integrins play an important role in the bone resorbing function of osteoclasts [13], [14]. Adherent-dependent cells, such as fibroblasts, epithelial cells, endothelial cells, and tumor cells, express several integrins and anchor to an appropriate extracellular matrix protein to survive, which leads to spreading, migration, differentiation, and tumor growth [15], [16], [17], [18]. Osteoclasts express α_v_β_3_ integrin, a vitronectin receptor, and tightly bind to bone surfaces via arginine-glycine-aspartic acid (RGD)-containing proteins, such as vitronectin [19], [20]. Osteoclasts that lack α_v_β_3_ integrin fail to activate c-Src, a tyrosine kinase associated with the cytoplasmic domain of RANK. c-Src is also involved in formation of ruffled borders [21] and sealing zones, and cell spreading of osteoclasts [22], [23]. However, the detailed relationships between cell adhesion, including integrin signaling and osteoclast differentiation have not been fully elucidated. To examine the roles of adherent conditions in osteoclast differentiation, Arai F et al. cultured osteoclast precursors in methylcellulose medium and found that osteoclast precursors failed to differentiate into osteoclasts due to a lack of adhesion signals [24]. In agreement with their results, we previously reported that interruption of cell adhesion to culture plates abrogated the differentiation of bone marrow-derived macrophages (BMMs) into osteoclasts, even in the presence of RANKL [25].

To suppress pathological bone resorption, investigators have sought potent inhibitors that target activated osteoclasts and several groups have isolated the RGD-containing disintegrin echistatin, a snake venom peptide of 49 amino acids isolated from viper *Echis carinatus* that inhibits α_v_β_3_ integrin-mediated cell adhesion [26]. It has also been shown that echistatin inhibits differentiation and the bone resorption function of osteoclasts, indicating essential roles of α_v_β_3_ integrin in osteoclast differentiation and function [27], [28], [29].

Despite accumulating evidence, the precise mechanisms by which cell adhesion signals are associated with osteoclast differentiation have not been fully elucidated. In the present study, we attempted to clarify the role of cell adhesion signaling in osteoclast differentiation *in vitro* using mouse BMMs (BMMs) under 2 different growth conditions; plastic cell culture plates (adherent condition) and the top surface of methylcellulose medium (non-adherent condition). We found that BMMs expressed RANK under the adherent condition, while that was expressed at a very low level under the non-adherent condition. Furthermore, RANK-overexpressing BMMs differentiated into functional osteoclasts even under the non-adherent condition. Our results suggest that cell adhesion is required for RANK expression in osteoclast precursors, which is essential for osteoclast differentiation.

## Materials and Methods

### Cytokines and chemicals

Human macrophage colony-stimulating factor (M-CSF) (Leukoprol®) and lipopolysaccharide (LPS) from *Escherichia coli* (O55∶B5) were purchased from Kyowa Hakko Kogyo (Tokyo, Japan) and Sigma-Aldrich (St. Louis, MO), respectively. Fetal bovine serum (FBS), α-modified minimum essential medium (αMEM), methylcellulose, antibiotics, and antimycotics were purchased from Invitrogen Life Technologies (Carlsbad, CA), Wako Pure Chemical Industries Ltd. (Osaka, Japan), Sigma-Aldrich, and Invitrogen Life Technologies, respectively. Recombinant human RANKL was kindly provided by Dr. Yongwon Choi (University of Pennsylvania). It is important to note that both human RANKL and mRANKL induce osteoclast differentiation (data not shown). Recombinant human transforming growth factor-β (TGF-β and recombinant mouse tumor necrosis factor-α (TNF-α were purchased from R&D Systems, Inc. (Minneapolis, MA). Rabbit polyclonal antibodies against phospho-IκB (#9241) and IκB (#9242) were purchased from Cell Signaling Technology, Inc. (Beverly, MA). Echistatin was purchased from Bachem Americas, Inc. (Torrance, CA).

### Preparation of BMMs

To prepare mouse BMMs, bone marrow cells were collected from the tibiae and femora of male mice (ddY strain, 4–6 weeks old), then cultured for 3 days in αMEM containing 10% FBS, TGF-β (1 ng/ml), and M-CSF (50 ng/ml) in cell culture dishes. The BMMs were removed from the dishes by treatment with trypsin EDTA and used as mouse osteoclast precursors. The Showa University Animal Care and Use Committee and Medical Ethics Committee gave approval for all of the procedures.

### Cell culture systems

**Figure 1:**
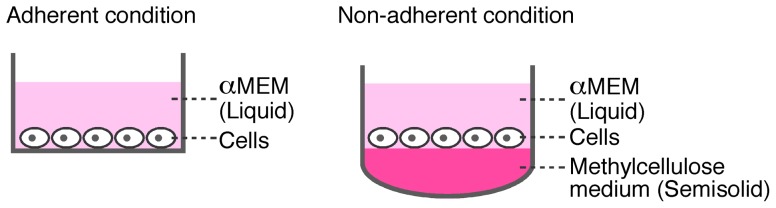
Schema of adherent and non-adherent cell culture systems used in this study. BMMs (1.25×10^5^/cm^2^) were cultured on plastic cell culture plates (*left*: adherent condition) or methylcellulose medium (*right*: non-adherent condition) in the presence of M-CSF (50 ng/ml), TGF-β (1.0 ng/ml), and RANKL (150 ng/ml) to examine osteoclast differentiation.

**Figure 2:**
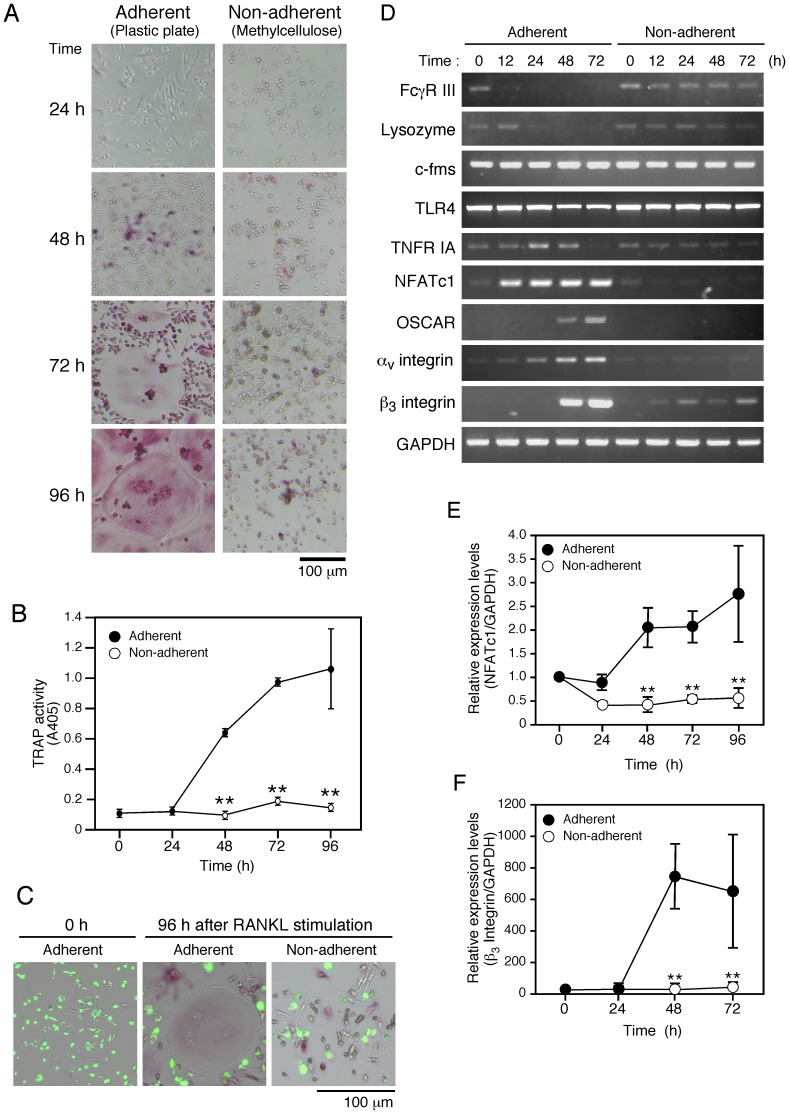
Osteoclast differentiation under adherent and non-adherent conditions. ***A*** *.* BMMs were cultured with M-CSF (50 ng/ml), TGF-β (1 ng/ml), and RANKL (150 ng/ml) under the adherent and non-adherent conditions for 24, 48, 72, or 96 hours, after which non-adherent cells were harvested and placed on plastic cell culture plates for 30 minutes, then fixed and stained for TRAP. Cells shown stained red are TRAP-positive cells. ***B*** *.* BMMs grown under adherent and non-adherent conditions were cultured in the presence of M-CSF (50 ng/ml), TGF-β (1 ng/ml), and RANKL (150 ng/ml) for 0, 24, 48, 72, or 96 hours. Subsequently, TRAP activity was determined. Data represent mean values of three independent experiments, with error bars indicating ± SD. ***C*** *.* FITC-conjugated zymosan particles were added to BMMs after 0 and 96 hours of culturing under the adherent and non-adherent conditions in the presence of M-CSF (50 ng/ml), TGF-β (1 ng/ml), and RANKL (150 ng/ml). One hour after zymosan addition, cells were washed with PBS and fixed, then the zymosan particles were visualized by UV illumination. Green dots indicate FITC-conjugated zymosan particles incorporated into cells. ***D*** *.* BMMs were cultured in the presence of M-CSF (50 ng/ml), TGF-β (1 ng/ml), and RANKL (150 ng/ml) under the adherent and non-adherent conditions for 0, 12, 24, 48, and 72 hours, after which mRNA expression levels were examined by RT-PCR. ***E and F*** *.* Relative mRNA expression levels of NFATc1 (***E***) and integrin β_3_ (***F***) at the indicated times under the adherent and non-adherent conditions in the presence of M-CSF (50 ng/ml), TGF-β (1 ng/ml), and RANKL (150 ng/ml), which were quantified using real-time RT-PCR. Data represent the mean values of three independent experiments, with error bars indicating ± SD. **P< 0.01 vs adherent condition at same time point.

To establish the non-adherent culture system, we placed 2 ml of 3% semisolid methylcellulose medium containing M-CSF (50 ng/ml), TGF-β (1 ng/ml), and RANKL (150 ng/ml) at the bottom of tubes (BD Falcon, CA) by centrifugation (200× g, 1 minute), then carefully loaded liquid αMEM containing M-CSF (50 ng/ml), TGF-β (1 ng/ml), RANKL (150 ng/ml), and BMMs (1.25×10^5^ cells/cm^2^) onto the methylcellulose medium. The cells sank down in the αMEM and settled on the top surface of the methylcellulose medium, without sinking further (***Fig. 1***). Cytokines and methylcellulose medium in the wells were well mixed using a glass stick for uniform diffusion. For the adherent condition, BMMs (1.25×10^5^) cells/cm^2^ were cultured in the presence of M-CSF (50 ng/ml), TGF-β (1 ng/ml), and RANKL (150 ng/ml) for 96 hours in cell culture plates (Corning, CA) for various time periods in the various experiments (***Fig. 2*** ***–*** ***6***).

**Figure 3:**
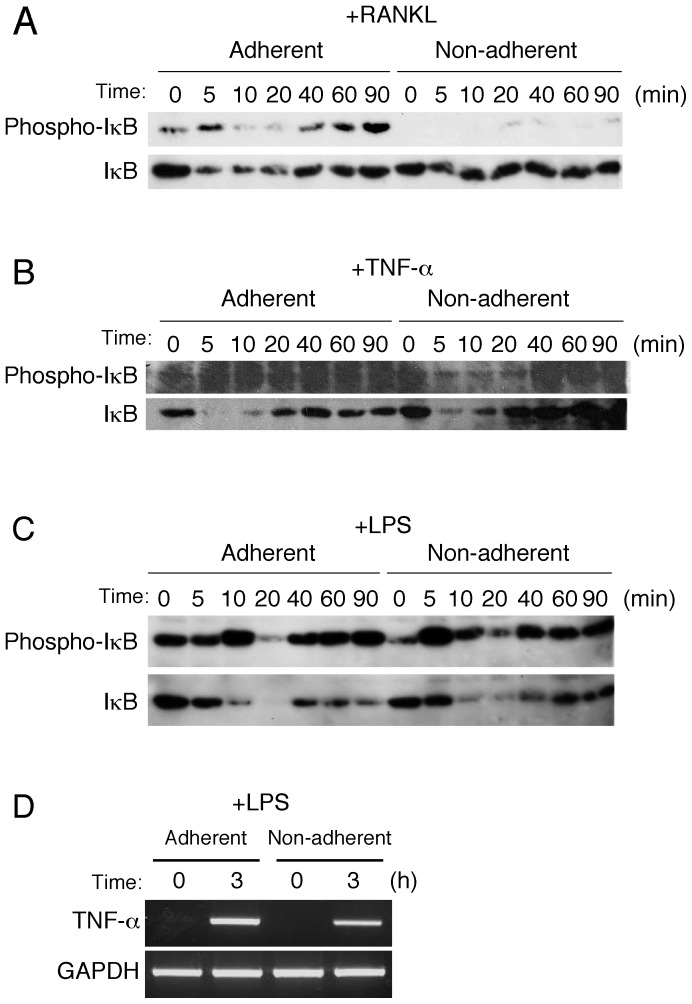
Effects of RANKL, TNF-α, and LPS on activation of NF-κB pathway. ***A–C*** *.* Cell lysates of BMMs stimulated with RANKL (150 ng/ml) (*A*), TNF-α (10 ng/ml) (*B*), or LPS (1 µg/ml) (***C***) for the indicated time periods under the adherent and non-adherent conditions were harvested, then phosphorylation and subsequent degradation of IκB were examined by immunoblot analysis. ***D*** *.* BMMs cultured under the adherent and non-adherent conditions were treated with LPS (1 µg/ml) for 3 hours, after which total RNA was extracted and TNF-α mRNA expression levels were examined by RT-PCR.

**Figure 4:**
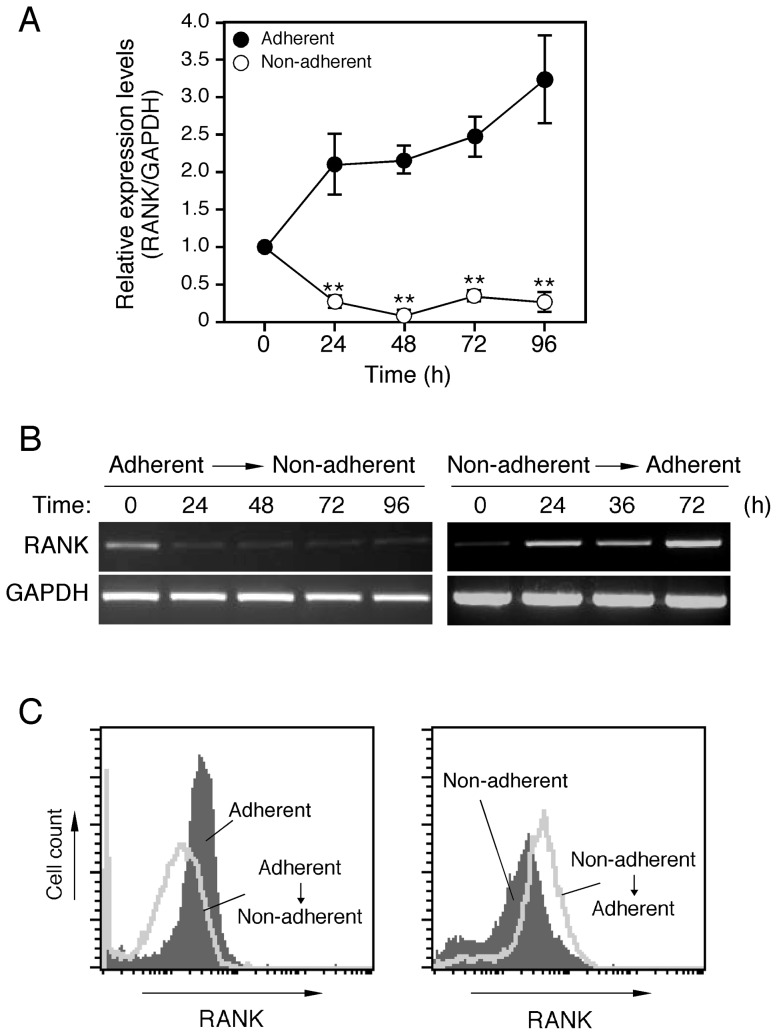
RANK expression levels under adherent and non-adherent conditions. ***A*** *.* BMMs under the adherent condition were harvested (0 h) and subsequently cultured again under adherent and non-adherent conditions in the presence or absence of M-CSF (50 ng/ml), TGF-β (1 ng/ml), and RANKL (150 ng/ml) for the indicated periods. Relative expression levels of RANK mRNA were determined by real time RT-PCR. Data represent mean values of three independent experiments, with error bars indicating ± SD. **P< 0.01 for adherent condition vs. non-adherent condition at the same time point. ***B*** *. Left panel*: BMMs grown under the adherent condition were transferred to the non-adherent condition and further cultured for the indicated time periods. *Right panel*: BMMs under the non-adherent condition were transferred to the adherent condition and further cultured for the indicated time periods. RANK mRNA expression was analyzed by RT-PCR. ***C***. *Left panel*: comparison of RANK protein expression levels between BMMs cultured under the adherent condition and those transferred to the non-adherent condition. *Right panel*: Comparison of RANK protein levels between BMMs cultured under the non-adherent condition and those transferred to the adherent condition. RANK protein expression on the surface of BMMs was analyzed by flowcytometry.

**Figure 5:**
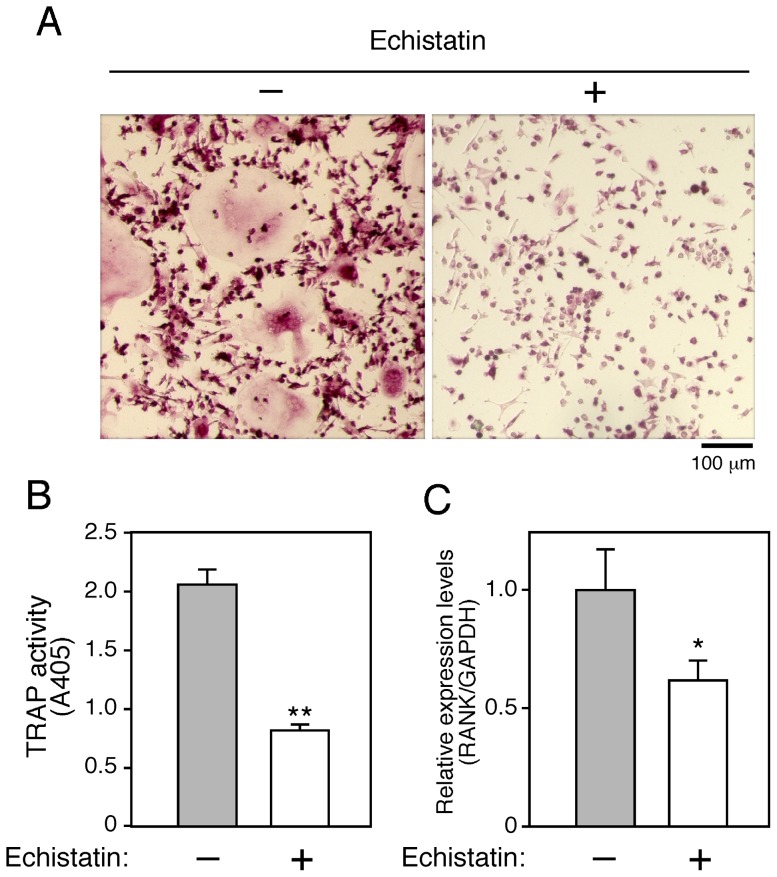
Effects of echistatin on osteoclast differentiation and RANK expression. ***A*** *.* BMMs were cultured in the presence of M-CSF (50 ng/ml), TGF-β (1 ng/ml), and RANKL (150 ng/ml) with or without echistatin (10^-8^ M). After culturing for 96 hours, cells were fixed and stained for TRAP (***A***), then TRAP activities were measured (***B***) and RANK expression levels evaluated (***C***). Data represent mean values of three independent experiments, with error bars indicating + SD. **P<0.01.

**Figure 6:**
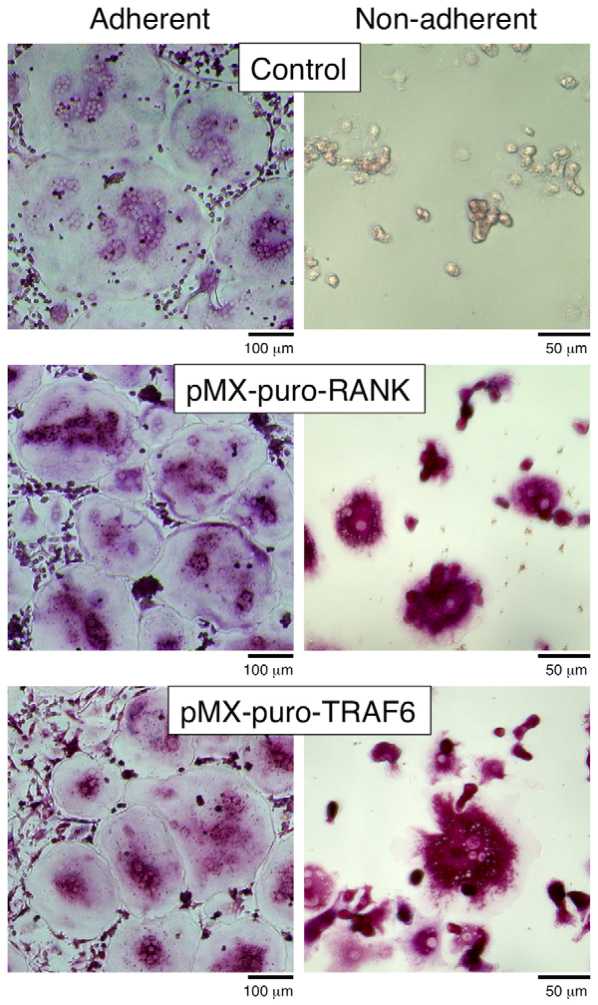
Osteoclast differentiation induced by forced expression of RANK and TRAF6 in BMMs under adherent and non-adherent condition. BMMs over-expressing RANK (transduced with pMX-RANK-puro vector) or TRAF6 (transduced with pMX-TRAF6-puro vector) were cultured with M-CSF (50 ng/ml), TGF-β (1 ng/ml), and RANKL (150 ng/ml) for 4 days under the adherent or non-adherent condition, then fixed and stained for TRAP. Cell under non-adherent condition were harvested and placed on cell culture plates for 1 hour to stain for TRAP.

### Detection of osteoclasts

After culturing BMMs for 72–96 hours, cells were fixed and stained for tartrate-resistant acid phosphatase (TRAP), a marker enzyme of osteoclasts, according to a previously reported method [30]. TRAP-positive multinucleated cells containing 3 or more nuclei were counted as osteoclasts.

### Measurement of TRAP activity

Cells in 96-well culture plates (adherent condition) or methylcellulose medium (non-adherent condition) were rinsed with PBS, then dissolved with 150 µl of lysis buffer (50 mM acetic acid buffer (pH 5.0) containing 1% sodium tartrate and 0.1% triton-X 100. The cell lysates were briefly sonicated to thoroughly dissolve the cell constituents on ice, then 30 µl of cell lysate was mixed with 100 µl of *p*-nitrophenyl phosphate solution [1 mg/ml in 50 mM acetic acid buffer (pH 5.0) containing 1% sodium tartrate] and incubated at 37°C for 30 minutes. After incubation, 70 µl of 1 M NaOH was added to the lysate and absorbance was measured at 405 nm.

### Forced expression of RANK and TRAF6 using retroviral vectors

Retrovirus packaging was performed by transfection of the retroviral vectors into BOSC23 cells using FuGENE6 (Roche), as previously reported [31]. Bone marrow cells were infected with the recombinant retroviruses in the presence of 50 ng/ml of M-CSF and 10 µg/ml of polybrene for 8 hours, and then the medium was replaced with fresh containing M-CSF (50 ng/ml), TGF-β (1 ng/ml), and RANKL (150 ng/ml). Cells were cultured for additional 48 hours in cell culture plates or tubes in methylcellulose medium. Those cultured in methylcellulose medium were collected by centrifugation and placed on cell culture plates to be stained for TRAP.

### Immunoblot analysis

BMMs were cultured in the presence of M-CSF (50 ng/ml) and TGF-β (1 ng/ml) in 60-mm diameter dishes or 3% methylcellulose medium in 14-ml polypropylene round-bottom tubes. Cells were stimulated with RANKL (150 ng/ml), TNF-α (100 ng/ml), and LPS (1 µg/ml) for various periods. Then, total cell lysates were isolated, separated by SDS-PAGE, and transferred onto Immobilon-P membranes (Millipore, Bedford, MA). The membranes were blocked with 5% non-fat milk in TBS-T (150 mM NaCl, 20 mM Tris, pH 7.4, 0.1% tween 20), then subjected to immunostaining with anti-phospho IκB (1∶1000) or anti-IκB (1∶1000) antibody, followed by the secondary horseradish peroxidase-conjugated antibody (1∶3000). The membranes were reacted using an enhanced chemiluminescence detection kit (Amersham Biosciences).

### Reverse transcription-polymerase chain reaction (RT-PCR) and quantitative RT-PCR

Total RNA from the cells in culture dishes (60-mm diameter) was prepared using TRIzol solution (Invitrogen, CA). First-stand cDNA was synthesized for PCR using Superscript III (Invitrogen) and subjected to amplification with Taq polymerase (Sigma-Aldrich) using the following specific PCR primers: mouse Fcγ-RIII, 5′-TGACACCCCATCCATCCTAT-3′(forward) and 5′-TATGCCATCAACCCTTAGCC-3′ (reverse); lysozyme, 5′-ACTGCTCAGGCCAAGGTCTA-3′ (forward) and 5′-GCCCTGTTTCTGCTGAAGTC-3′ (reverse); toll-like receptor (TLR) 4, 5′-ACCTGGCTGGTTTACACGTC-3′ (forward) and 5′-CAGGCTGTTTGTTCCCAAAT-3′ (reverse); NFATc1, 5′-TCATCCTGTCCAACACCAAA-3′ (forward) and 5′-TTGCGGAAAGGTGGTATCTC-3′ (reverse); osteoclast associated receptor (OSCAR), 5′-ACTCCTGGGATCAACGTGAC-3′ (forward) and 5′-GATAGCACATAGGGGGCAGA-3′ (reverse); receptor activator of NF-κB (RANK), 5′- TGCAGCTCAACAAGGATACG -3′ (forward) and 5′- ACCATCTTCTCCTCCCGAGT -3′ (reverse); integrin α_V_, 5′-ACACTTTGGGCTGTGGAATC-3′ (forward) and 5′-CGCCACTTAAGAAGCACCTC-3′ (reverse); integrin β_3_, 5′-GACCACAGTGGGAGTCCTGT-3′ (forward) and 5′-GAGGGTCGGTAATCCTCCTC-3′ (reverse); and c-fms, Glyceraldehyde-3-phosphate dehydrogenase (GAPDH), 5′-GAAGGTCGGTGTGAACGGATTTGGC-3′ (forward) and 5′-CATGTAGGCCATGAGGTCCAACAC-3′ (reverse).

RANK, NFATc1, and integrin β_3_ mRNA expressions were quantified using a Taqman® Real-time PCR system (Applied Biosystems Japan, Tokyo, Japan) with mouse RANK (Assay ID: Mm00437135_m1), human RANK (Assay ID: Hs00187192_m1), mouse NFATc1 (Assay ID: Mm00479445_m1), mouse integrin β_3_ (Assay ID: Mm00443980_m1), mouse GAPDH (Assay ID: Mm03302249_g1), and human GAPDH (Assay ID: Hs02758991_g1) probes, which were provided by the manufacturer.

### Flow cytometry

To detect RANK on BMMs, cells were stained with biotinylated anti-mouse RANK antibody (BAF692: R&D Systems Inc.). After washing with PBS twice, the cells were incubated with phycoerythrin (PE)-conjugated anti-rat IgG (Beckman Coulter). The expression of RANK on the cells was analyzed using a FACSCalibur (BD Pharmingen).

### Statistical analyses

Differences in data between groups were analyzed using Student's *t* test and two-way analysis of variance (ANOVA). ANOVA was followed by the Bonferroni post-hoc multiple comparison test when appropriate, and all data are presented as the mean ± SD. Probability values less than 0.05 were considered significant. Statistical analyses were conducted with STATISTICA Advanced 06J (StatSoft Japan Inc., Tokyo, Japan). Results are representative of more than three individual experiments.

## Results

### Osteoclast precursors failed to differentiate into osteoclasts under non-adherent condition

BMMs cultured on plastic cell culture plates (adherent condition) in the presence of RANKL differentiated into TRAP-positive multinucleated osteoclasts within 72 hours (***Fig. 2A, B***). On the other hand, BMMs cultured on methylcellulose medium (non-adherent condition) failed to differentiate into osteoclasts, though a small number of mononuclear osteoclasts appeared (***Fig. 2A, B***). In addition, the non-adherent condition did not induce anoikis among the BMMs (***Fig. S1***) and the methylcellulose compound itself had no effects on osteoclast differentiation (***Fig. S2***). To examine whether BMMs cultured under the non-adherent condition retained macrophage function, they were cultured in the presence of FITC-conjugated zymosan particles to evaluate their phagocytic function. Mature osteoclasts that differentiated under the adherent condition did not incorporate the zymosan particles (***Fig. 2C***), while BMMs that failed to differentiate into osteoclasts incorporated those particles (***Fig. 2C***), suggesting that BMMs under the non-adherent condition retained the function of macrophages. To further examine the characteristics of cells cultured under both conditions, we analyzed the expressions of macrophage and osteoclast marker genes (***Fig. 2D***). BMMs stimulated with RANKL under the adherent condition showed upregulation of osteoclast marker genes, such as NFATc1, α_v_ and β_3_ integrins, and OSCAR, whereas macrophage specific genes such as FcγR III and lysozyme were downregulated in a time-dependent manner (***Fig. 2D, E***). Under the non-adherent condition, the expressions of osteoclast marker genes were not up-regulated in BMMs (***Fig. 2D, E***), while those of macrophage marker genes remained (***Fig. 2D***), some of which were also confirmed by quantitative RT-PCR (***Fig. 2E, F***). In addition, the cells expressed c-Fms, a receptor of M-CSF, TLR4, a receptor of LPS, and type I TNF receptor (TNFR IA) throughout the culture period under both the adherent and non-adherent conditions (***Fig. 2D***).

### RANKL failed to activate NF-κB-mediated signaling pathways under non-adherent condition

To explore the mechanism by which BMMs did not differentiate into osteoclasts even in the presence of RANKL under the non-adherent condition, we examined the activation of intracellular signaling molecules including IκB. Under the adherent condition, RANKL induced the phosphorylation of IκB within 5 minutes, followed by the degradation of IκB within 10 minutes. After 40 minutes, the expression level of IκB was recovered, suggesting that cell signaling was transduced through NF-κB mediated pathways under the adherent condition (***Fig. 3A***). In contrast, under the non-adherent condition, RANKL poorly induced the phosphorylation and following degradation of IκB, while the protein level of IκB was maintained throughout the experimental period (***Fig. 3A***). Next, we examined the effect of TNF-αon activation of the intracellular signaling pathway, as TNF-α is known to induce osteoclastogenesis independent of RANKL [32] TNF-α induced phosphorylation following the degradation of IκB under both the adherent and non-adherent conditions (***Fig. 3B***). Since BMMs continuously expressed TLR4 under both conditions (***Fig. 2D***), we also examined the effects of LPS on activation of intracellular signaling and TNF-α mRNA expression (***Fig. 3C***). LPS induced phosphorylation and degradation of IκB, and subsequent TNF-α mRNA expression equally under both conditions (***Fig. 3D***). These results suggest that RANKL failed to activate the intercellular signaling pathways under the non-adherent condition, though intercellular signaling pathways are normally function in BMMs.

### Cell adhesion signaling is required for RANK expression in BMMs

Since RANKL failed to activate the intercellular signaling pathways in BMMs under the non-adherent condition, we analyzed RANK expression in osteoclast precursors. When BMMs were stimulated with RANKL under the adherent condition, the expression level of RANK mRNA increased in a time-dependent manner (***Fig. 4A***). However, under the non-adherent condition, that was markedly decreased even in the presence of RANKL (***Fig. 4A***). When BMMs under the adherent condition were transferred to the non-adherent condition, the RANK mRNA expression level was decreased within 24 hours (***Fig. 4B*** ***, left panel***). In contrast, when BMMs under the non-adherent condition were transferred to the adherent condition, that expression was increased within 24 hours (***Fig. 4B*** ***, right panel***). We also analyzed the production of RANK protein using flow cytometry (***Fig. 4C***). Transfer of BMMs from the non-adherent to adherent condition induced the expression of RANK protein, while transfer from the adherent to non-adherent condition reduced it (***Fig. 4C***). These results suggest that cell adhesion signaling in osteoclast precursors upregulates the expression of RANK in BMMs. In addition, human peripheral blood monocytes (hPBMCs) also differentiated into osteoclasts under the adherent condition and failed to do so under non-adherent condtion (***Fig. S3A, B***). The expression level of RANK mRNA in PBMCs under the adherent condition was higher than that under the non-adherent condition (***Fig. S3C***).

### Blockade of integrin-mediated signaling inhibited RANK expression

We also examined whether integrin-mediated signaling participates in regulation of RANK expression in osteoclast precursors using echistatin, an RGD-containing snake venom, which inhibits fusion of mononuclear osteoclast precursors, as well as the migration and activity of osteoclasts by inhibiting α_v_β_3_ integrins [27], [29]. Addition of echistatin to cultures of BMMs under the adherent condition strongly inhibited the formation of osteoclasts (***Fig. 5A, B***), while RANK expression was also suppressed in those cells *(* ***Fig. 5C*** *)*. These results suggest that α_v_β_3_ integrin may be involved in the induction of RANK expression, which in turn accelerates osteoclast differentiation.

### Forced expression of RANK or TRAF6 restored osteoclast differentiation under non-adherent condition

To confirm that the decreased expression of RANK in BMMs is the main reason for their inability to undergo osteoclastic differentiation under the non-adherent condition, RANK and TRAF6, the major adaptor molecules of RANK, were individually over-expressed in BMMs using retroviral vectors encoding RANK cDNA (pMX-puro-RANK). Under the adherent condition, forced expression of either RANK or TRAF6 in BMMs induced differentiation into osteoclasts in both the absence and presence of RANKL (***Fig. 6***). Moreover, RANK- and TRAF6-overexpressing BMMs differentiated into TRAP-positive multinucleated cells even under the non-adherent condition (***Fig. 6***). These results suggest that the impaired osteoclast differentiation observed under the present non-adherent condition was due to an absence of RANK-dependent signaling by osteoclast precursors.

## Discussion

The extracellular matrix is essential for embryonic development, postnatal homeostasis, and immune system activities [33], [34], [35]. Cell adhesion signals activate various intracellular signaling systems and are required for cellular differentiation, proliferation, and other functions. In osteoblasts, interaction between α_2_ integrin and collagen is required for activation of *cbfa1*, an essential transcription factor for osteoblasts, and the following induction of osteoblast-specific gene expression [36]. In a study of osteoclast differentiation, Miyamoto *et al*. found that cell adhesion is required, though the reason has not been fully elucidated [37]. In the present study, we addressed these questions and found that cell adhesion signaling regulates RANK expression in osteoclast precursors.

BMMs failed to differentiate into osteoclasts when cultured on methylcellulose medium, to which the cells did not adhere. In addition, we also found that BMMs scarcely adhered to collagen gel and the expression level of RANK level in those cultures was downregulated as compared to cultures on the plastic plates (***Fig. S4***). From these results, we speculated that the cellular characteristics of BMMs under non-adherent conditions are different from those under adherent conditions. Indeed, the expression of lysozyme mRNA, a typical enzyme related to macrophages, was maintained in BMMs under the non-adherent condition, whereas that of NFATc1, an essential transcription factor for osteoclast differentiation, was not upregulated in BMMs in response to RANKL under the non-adherent condition. Thus, under non-adherent conditions, BMMs do not differentiate into osteoclasts even in the presence of RANKL.

In addition to RANKL, TNF-α and LPS are known to induce NF-κB activation by inducing the phosphorylation and following degradation of IκB [38]. We found that these factors induced intracellular signaling by activation of IκB in BMMs under both the non-adherent and adherent conditions. However, RANKL failed to transduce intracellular signaling under the non-adherent condition. Thus, these findings imply an *in vivo* situation in which osteoclast precursors are able to respond to immune stimuli without adhesion signals, though they require adhesion to bone and osteoblasts for differentiation into osteoclasts.

Since RANKL failed to transduce signals in BMMs under the non-adherent condition, we speculated that non-adherent BMMs lacked RANK. Indeed, the level of RANK expression in non-adherent BMMs was lower than that compared to adherent BMMs. On the other hand, RANK expression level was dramatically increased after transferring BMMs from the non-adherent to adherent condition. Furthermore, echistatin inhibited osteoclast differentiation under the adherent condition. Together, these results suggest that α_v_β_3_ integrins mediate signals that induce RANK expression.

TRAF6 is a crucial adaptor protein in the RANK-mediated signaling pathway for induction of osteoclast differentiation [39], [40], [41]. Even under the present non-adherent condition, both RANK- and TRAF6-overexpressing BMMs differentiated into osteoclasts in response to RANKL, and formed resorption pits on dentin slices. These results also support the notion that adhesion is critically involved in the determination of cell fate of osteoclast precursors via mechanisms that determine RANK expression.

In conclusion, our findings indicate that cell adhesion and subsequent induction of RANK in osteoclast precursors is essential for differentiation into osteoclasts following RANKL stimulation. These results may explain, at least in part, the regulatory system related to osteoclast localization on bone surfaces.

## Supporting Information

Figure S1 **Viabilities of BMMs cultured under adherent and non-adherent conditions.** BMMs were cultured in plastic cell culture plates (*left*) or on methylcellulose medium (*right*) for 24 hours, then fixed and stained with DAPI to visualize the nuclei. Nuclei in both cells were normal, suggesting that they did not undergo apoptosis.
(TIF)

Figure S2 **Effects of methylcellulose compound on osteoclast differentiation under adherent condition.** BMMs were cultured in the presence of M-CSF (50 ng/ml), TGF-β (1 ng/ml), and RANKL (150 ng/ml) with or without methylcellulose (2 mg/ml) for 96 hours on plastic cell culture plates, then fixed and stained for TRAP. TRAP-positive cells appear red.
(TIF)

Figure S3 **Human osteoclast differentiation under adherent and non-adherent conditions.** Human CD14^+^ cells as osteoclast precursors were collected from whole blood samples obtained from healthy donors using Lympholyte-H® (Cedarlane laboratories, Ontario, Canada). Cells were cultured in the presence of M-CSF (50 ng/ml), TGF-β (1 ng/ml), and RANKL (30 ng/ml) under adherent and non-adherent conditions for 96 hours (1.25×10^5^ cells/cm^2^). After staining for TRAP (***A***), osteoclasts containing more than 3 nuclei were counted (***B***) and RANK expression levels were evaluated using quantitative RT-PCR (***C***). Error bars represent the mean ± SD. **P< 0.01 for adherent condition vs. non-adherent condition. All procedures were approved by the Showa University Medical Ethics Committee.
(TIF)

Figure S4 **Expression levels of RANK in BMMs cultured on collagen gel as a non-adherent condtion.** BMMs were cultured in the presence of M-CSF (50 ng/ml), TGF-β (1 ng/ml), and RANKL (150 ng/ml) on plastic cell culture plates, methylcellulose medium, or 3 mg/ml semisolid collagen gel (Nitta gelatin, Tokyo, Japan: non-adherent condition) for 72 hours, then the relative expression levels of RANK mRNA were quantified using quantitative RT-PCR. Data represent the mean values of 3 independent experiments, with error bars indicating ± SD. **P< 0.01 vs. adherent condition at the same time point.
(TIF)
