# Supplementary figures and images for: Cell Adhesion Signaling Regulates RANK Expression in Osteoclast Precursors
Source: PLoS One. 2012 Nov 6;7(11):e48795. doi: 10.1371/journal.pone.0048795 (PMC3490906; doi:10.1371/journal.pone.0048795)

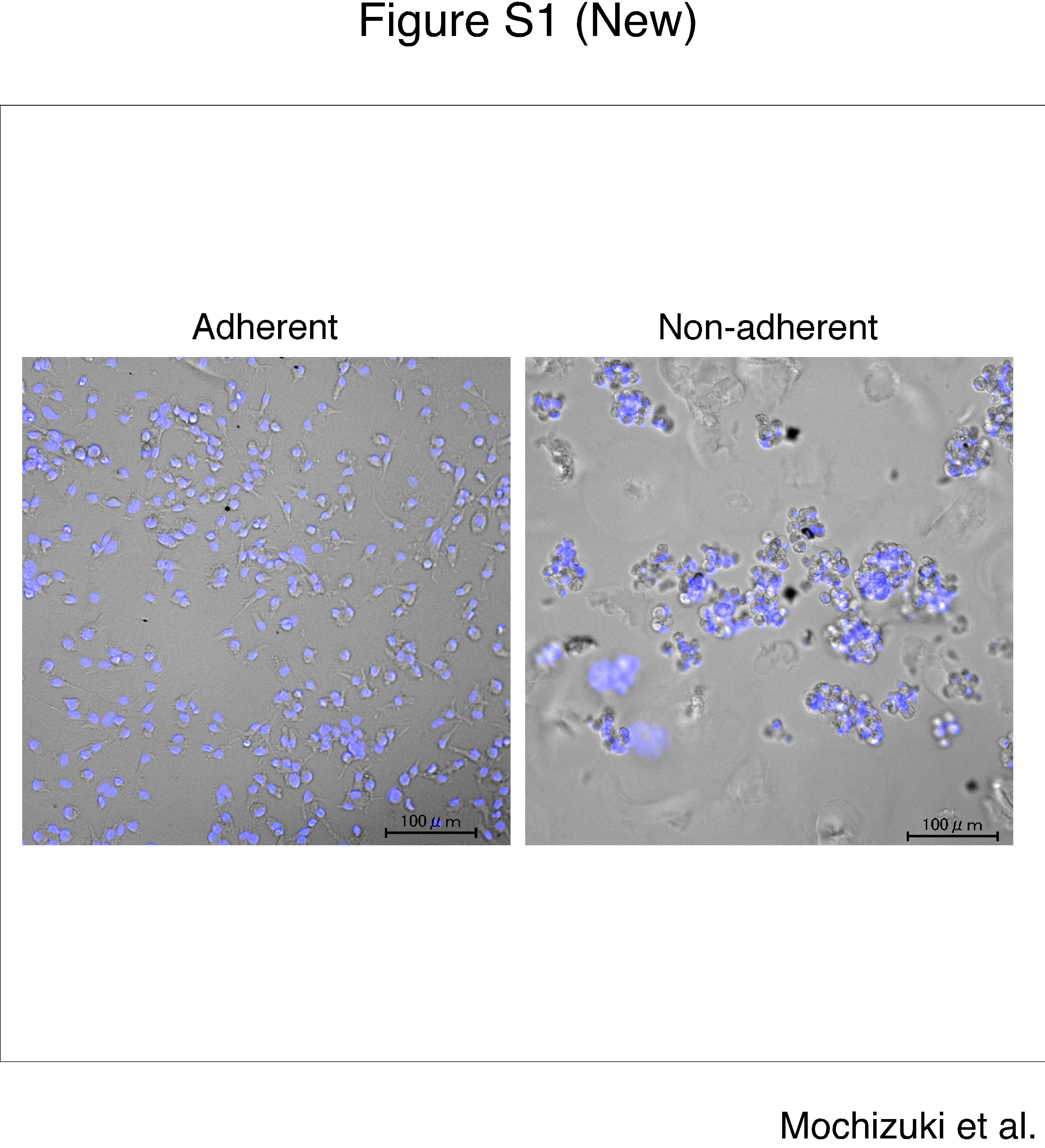

Supplement: Figure S1 — Viabilities of BMMs cultured under adherent and non-adherent conditions. BMMs were cultured in plastic cell culture plates (left) or on methylcellulose medium (right) for 24 hours, then fixed and stained with DAPI to visualize the nuclei. Nuclei in both cells were normal, suggesting that they did not undergo apoptosis. (TIF) [file pone.0048795.s001.tif]

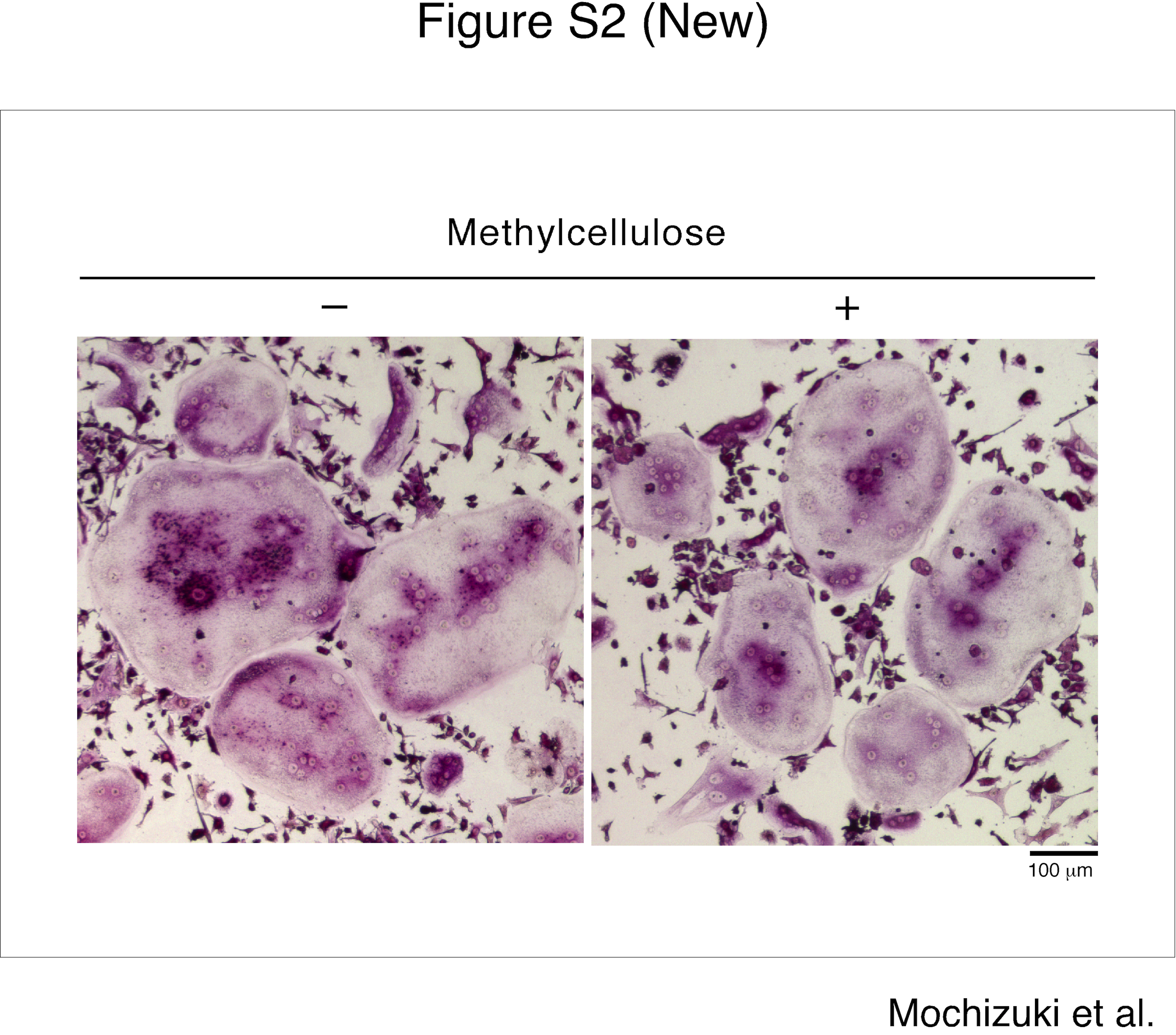

Supplement: Figure S2 — Effects of methylcellulose compound on osteoclast differentiation under adherent condition. BMMs were cultured in the presence of M-CSF (50 ng/ml), TGF-β (1 ng/ml), and RANKL (150 ng/ml) with or without methylcellulose (2 mg/ml) for 96 hours on plastic cell culture plates, then fixed and stained for TRAP. TRAP-positive cells appear red. (TIF) [file pone.0048795.s002.tif]

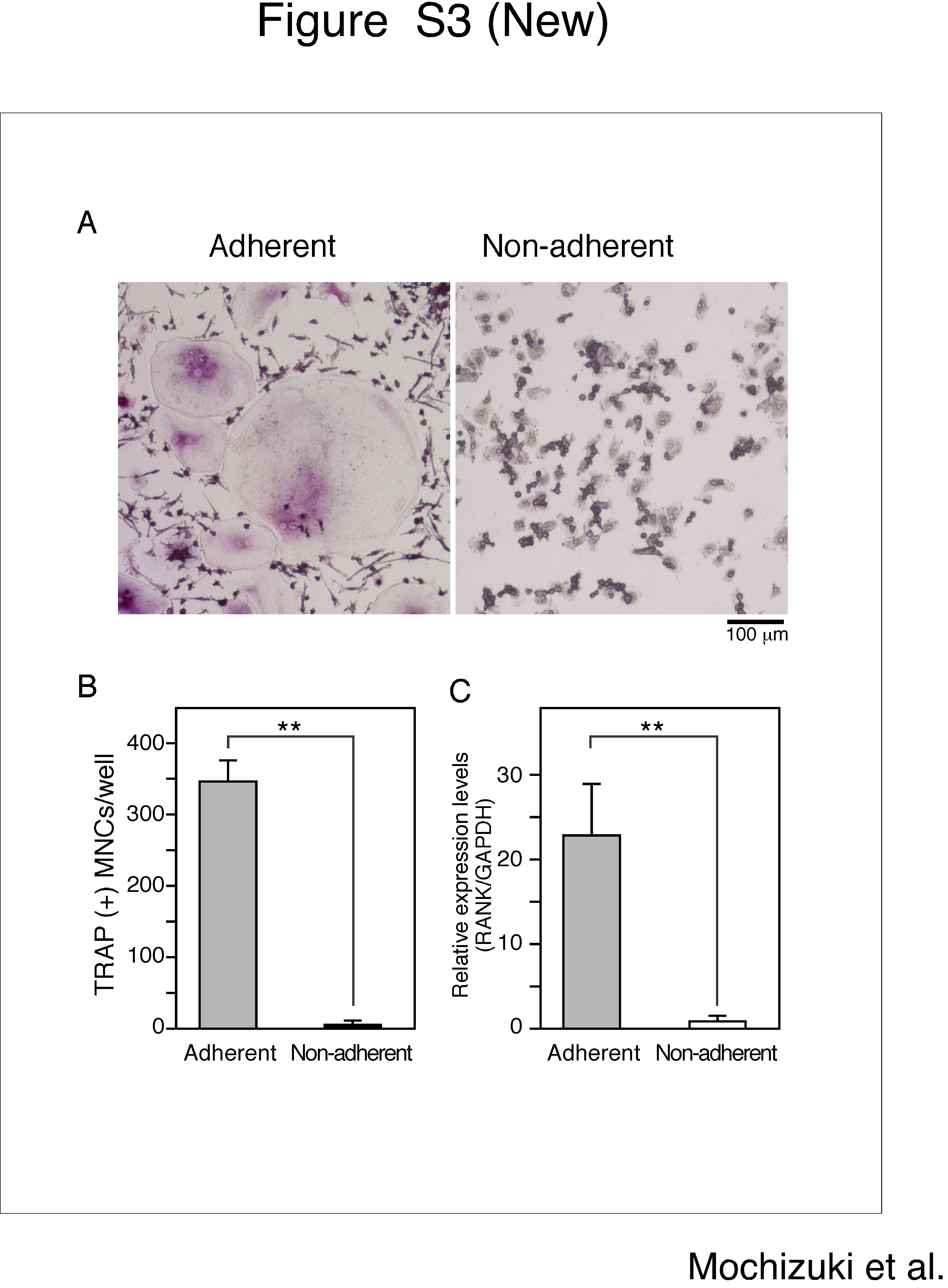

Supplement: Figure S3 — Human osteoclast differentiation under adherent and non-adherent conditions. Human CD14+ cells as osteoclast precursors were collected from whole blood samples obtained from healthy donors using Lympholyte-H® (Cedarlane laboratories, Ontario, Canada). Cells were cultured in the presence of M-CSF (50 ng/ml), TGF-β (1 ng/ml), and RANKL (30 ng/ml) under adherent and non-adherent conditions for 96 hours (1.25×10^5^ cells/cm2). After staining for TRAP (A), osteoclasts containing more than 3 nuclei were counted (B) and RANK expression levels were evaluated using quantitative RT-PCR (C). Error bars represent the mean ± SD. **P< 0.01 for adherent condition vs. non-adherent condition. All procedures were approved by the Showa University Medical Ethics Committee. (TIF) [file pone.0048795.s003.tif]

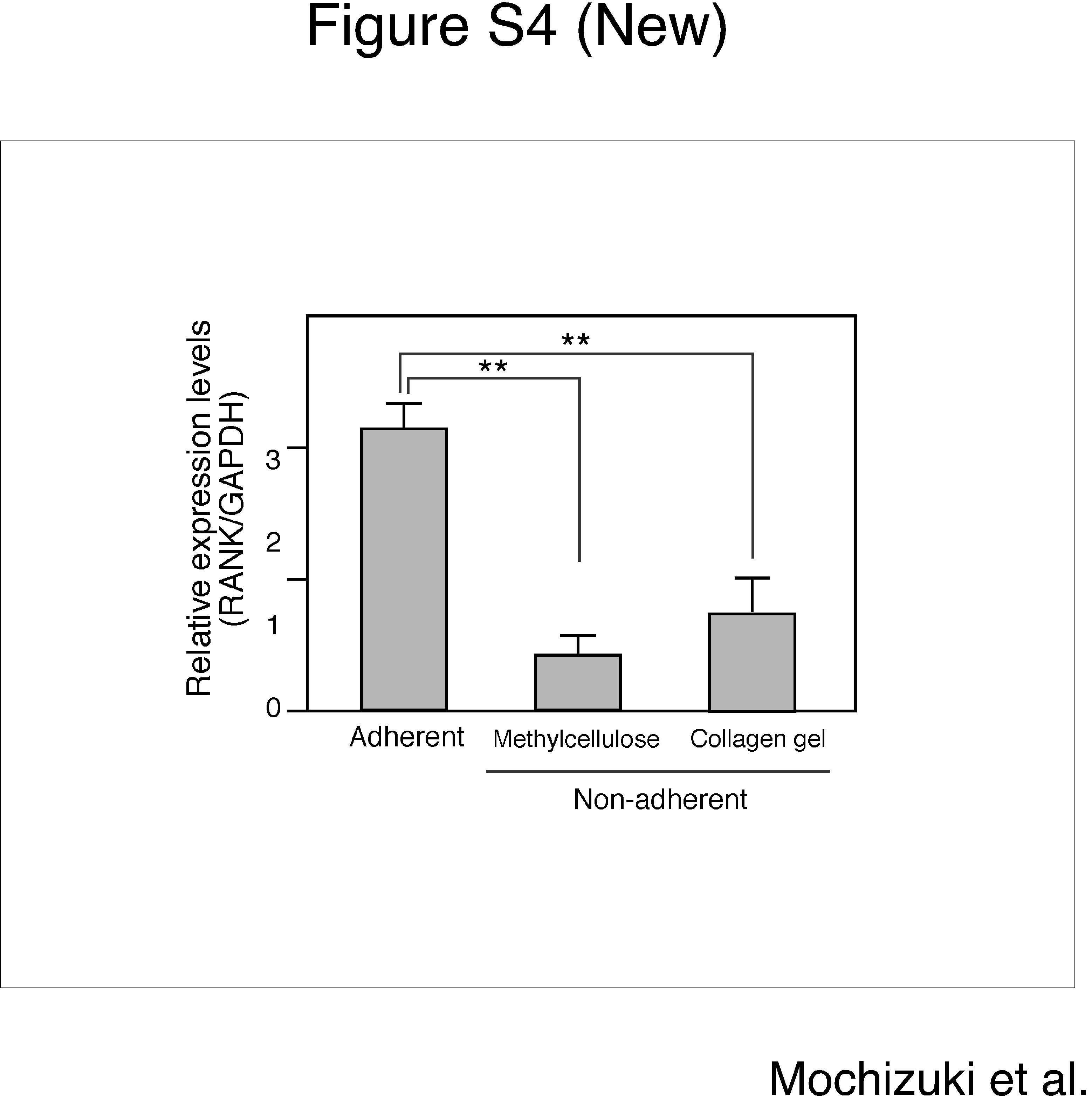

Supplement: Figure S4 — Expression levels of RANK in BMMs cultured on collagen gel as a non-adherent condtion. BMMs were cultured in the presence of M-CSF (50 ng/ml), TGF-β (1 ng/ml), and RANKL (150 ng/ml) on plastic cell culture plates, methylcellulose medium, or 3 mg/ml semisolid collagen gel (Nitta gelatin, Tokyo, Japan: non-adherent condition) for 72 hours, then the relative expression levels of RANK mRNA were quantified using quantitative RT-PCR. Data represent the mean values of 3 independent experiments, with error bars indicating ± SD. **P< 0.01 vs. adherent condition at the same time point. (TIF) [file pone.0048795.s004.tif]
